# Solid-State Dewetting of Thin Au Films for Surface Functionalization of Biomedical Implants

**DOI:** 10.3390/ma16247524

**Published:** 2023-12-06

**Authors:** Aliya Sharipova, Ivan Zlotver, Alejandro Sosnik, Eugen Rabkin

**Affiliations:** 1 Department of Materials Science and Engineering, Technion–Israel Institute of Technology, Haifa 3200003, Israel; aliya.sharipova@ikts.fraunhofer.de (A.S.); ivan@campus.technion.ac.il (I.Z.); sosnik@technion.ac.il (A.S.); 2 Department of Bio- and Nanotechnology, Fraunhofer Institute for Ceramic Technologies and Systems IKTS, 01277 Dresden, Germany

**Keywords:** biomaterials, titanium alloys, thin films, wetting, surface functionalization

## Abstract

Biomaterial-centered infections of orthopedic implants remain a significant burden in the healthcare system due to sedentary lifestyles and an aging population. One approach to combat infections and improve implant osteointegration is functionalizing the implant surface with anti-infective and osteoinductive agents. In this framework, Au nanoparticles are produced on the surface of Ti-6Al-4V medical alloy by solid-state dewetting of 5 nm Au film and used as the substrate for the conjugation of a model antibiotic vancomycin via a mono-thiolated poly(ethylene glycol) linker. Produced Au nanoparticles on Ti-6Al-4V surface are equiaxed with a mean diameter 19.8 ± 7.2 nm, which is shown by high-resolution scanning electron microscopy and atomic force microscopy. The conjugation of the antibiotic vancomycin, 18.8 ± 1.3 nm-thick film, is confirmed by high resolution-scanning transmission electron microscopy and X-ray photoelectron spectroscopy. Overall, showing a link between the solid-state dewetting process and surface functionalization, we demonstrate a novel, simple, and versatile method for functionalization of implant surfaces.

## 1. Introduction

Solid-state dewetting (DW) of thin metallic films is a spontaneous agglomeration of as-deposited thin films into separate islands or particles during heat treatments well below the melting point of metal [1]. This process is undesirable in microelectronics because spontaneous agglomeration of metallic contacts at elevated temperatures leads to device failure. However, the DW process might be beneficial when spontaneous agglomeration of metallic films into particles is desired, and the term “dewetting engineering” was coined to describe the fabrication of metal nanoparticles (NPs) for functional applications employing the DW process [2].

Recently, several groups have proposed the use of solid-state DW to obtain nanoparticle (NP)-decorated surfaces for biomedical applications such as biomedical diagnostics [3], biosensing [4,5,6], and implant surface functionalization [7,8]. Specifically, cellulose paper decorated with silver (Ag) NPs was suggested for rapid separation and label-free detection of diverse biomolecules in body fluids [3]. Au-Ag alloy nanoislands produced by solid-state DW showed outstanding localized surface plasmon resonance sensitivity and improved anti-degradation performance for label-free biochemical detection [4]. Au NPs on the epoxy pillars were successfully used for label-free plasmonic detection of deoxyribonucleic acid [5]. Highly sensitive surface-enhanced Raman scattering sensors based on Ag-silicon nanospheres were suggested for the potential detection of pharmaceutical intermediates, e.g., p-thiocresol [6]. Titanium (Ti)-based medical alloys such as Ti-6Al-4V decorated with titanium oxide (TiO_2_) nanopimples produced by alkalinity-activated DW showed strengthening of in vivo bone–implant interfacial bonding [7]. Finally, TiO_2_ films decorated by DW-produced Au NPs showed antibacterial activity against *Veillonella parvula* and *Neisseria sicca* species associated with oral diseases [9]. However, to the best of our knowledge, no reports on the application of solid-state DW processes to functionalize implant surfaces with drugs are available in the literature.

Implant surface functionalization with drugs is one of the strategies to combat bacterial biomaterial-centered infections of orthopedic implants, which remains a significant burden in the healthcare system and has shown an increasing trend over the last decades due to sedentary lifestyles and an aging population [10,11]. Functionalization of Ti-6Al-4V surfaces with the antibiotic vancomycin (VH) using plasma deposition effectively inhibits bacterial adhesion and reduces the inflammatory response in bacterially challenged host tissues [12]. Similarly, the modification of Ti-6Al-4V surfaces with TiO_2_ nanotubes and loading them with a gentamicin-VH mixture provides local bactericidal properties against Gram-positive *Staphylococcus aureus* accompanied with good in vivo cytocompatibility and osteointegration [13]. Modification of Ti-6Al-4V surface by a calcium titanate layer enables conjugation and steady in vitro release of bisphosphonate drug that prevents osteoporosis [14,15]. An alternative approach to implant functionalization involves the conjugation of anti-infective and/or osteoinductive agents to chemically activated Au NPs [16]. However, Au NP deposition onto an implant surface by conventional methods lacks control over NP distribution and adhesion. Some methods suffer from poor reproducibility, while others are implant material-specific [17,18]. In particular, the electrochemical deposition is restricted to conductive materials [19,20]. Chemical reduction methods are simple but employ toxic and expensive reagents [21,22]. Hydrothermal methods lack control over the process parameters, which adversely affects the reproducibility of the final nanostructures [23,24]. In this respect, employing the solid-state DW process to fabricate NPs on the implant surface resolves several problems: this method is insensitive to the composition of the implant material, does not require complex reagents, and enables control over the size of NPs by varying the thickness of the deposited Au film [1,2]. Recently, Sharipova et al. described the mechanisms of solid-state DW of thin Au films on the rough surface of the oxidized Ti-6Al-4V alloy [25], and suggested that Au NPs fabricated by DW can be used for implant surface functionalization with drugs.

**Figure 1:**
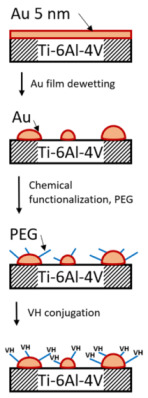
Schematic of the Ti-6Al-4V surface functionalization with antibiotic vancomycin via Au NPs fabricated by solid-state dewetting of thin Au film.

Here, we report on the functionalization of a Ti-6Al-4V surface with the broad-spectrum antibiotic VH via novel chemically activated Au NPs prepared by solid-state DW (Figure 1). We hypothesized that the DW process will produce homogeneously distributed Au NPs on the Ti-6Al-4V surface, and the following conjugation of antibiotic molecules would create a bactericidal layer on the alloy surface, which could inhibit bacterial proliferation and prevent biofilm formation. In this framework, Au NPs prepared by the solid-state DW would provide novel functional properties to the Ti-6Al-4V implant surface.

## 2. Material and Methods

### 2.1. Preparation of Ti-6Al-4V Decorated with Au NPs by Solid-State DW Process

High-purity Ti alloy with an extra low concentration of interstitial elements (Ti-6Al-4V; Ti90/Al6/V4 ELI Grade 23) was purchased in rod shape (Ø 10 mm) from Advent Research Materials Ltd. (Eynsham, Oxford, UK, OX29 4JA), cut into ~1 mm thick disks, and handled for Au film deposition. Prior to the deposition, Ti-6Al-4V samples were cleaned in subsequent ultrasound baths of acetone, methanol, and isopropanol. Electron-beam deposition (Temescal BJD 1800, Edwards Vacuum GmbH (currently Ferrotec)/Ferrotec Europe GmbH, Unterensingen, Germany) of 5 nm thick Au film was performed in a vacuum chamber with a base pressure of 4.7 × 10^−7^ Torr at room temperature (RT) at a rate ~0.1 nm/s. Such film thickness was chosen to decrease the following DW temperature [1]. Samples with deposited Au films were annealed in a rapid thermal annealing furnace (RTA; ULVAC-RIKO MILA-5000-P-N, Methuen, MA, USA) at 200 °C for 3 h to cause full dewetting of the deposited film. The annealing was performed in forming gas flow (Ar + 10 vol. % H_2_) to avoid simultaneous Ti-6Al-4V oxidation with solid-state DW. The selected annealing regime prevents grain coarsening and/or phase changes of Ti-6Al-4V, which typically occur above 550 °C [26,27]. The combination of relatively low annealing temperature with ultralow film thickness resulted in fully DW-ed Au NPs with no structural changes of Ti-6Al-4V. A low annealing temperature also preserves the Ti-6Al-4V surface topography, which is critical for bone cell attachment and proliferation [28,29].

### 2.2. Functionalization of Ti-6Al-4V Surface Decorated with Au NPs

Chemically functionalized poly(ethylene glycol) (PEG) linker was conjugated to Au NPs using a heterobifunctional thiol-polyethylene-acid (HS-PEG-COOH; Laysan Bio Inc. Arab, AL, USA). For this, Ti-6Al-4V decorated with Au NPs were immersed in an HS-PEG-COOH aqueous solution (0.5 mM) at RT for 2 h and washed 3 times with double distilled water (DDW; Milli-Q Barnstead Smart2Pure 3 L UV/UF water purification system; Thermo Fisher Scientific Inc., Waltham, MA, USA). Chemical activation of the carboxyl-PEG groups with the following coupling to amine groups of VH hydrochloride (VH; C_66_H_75_Cl_2_N_9_O_24_·HCl, STREM Chemicals, Newburyport, MA, USA) was performed using the N-ethyl-N′-(3-(dimethylamino)propyl)carbodiimide/N-hydroxysuccinimide (EDC/NHS, Glentham Life Science Ltd., Wilshire, UK, and Chem-Impex International, Wood Dale, IL, USA, respectively) condensation reaction in water. EDC and NHS were dissolved and covered the PEGylated Au NPs on Ti-6Al-4V for 30 min at RT, providing active sites on PEG moieties that undergo an amidation reaction with VH. Samples were washed three times in DDW to remove reagent residues. Then, the samples were immersed in DDW with the drug dissolved in ten-fold molar excess with respect to the PEG moieties to ensure conjugation in all the reactive sites. The samples were incubated overnight, washed three times to remove the unbound drug, and kept in a dry environment (desiccator with silica gel) at RT.

### 2.3. Characterization of Microstructure, Roughness, and Chemical Composition

The microstructure, roughness, and chemical composition of the samples were characterized using high-resolution scanning electron microscopy (HR-SEM; Ultra+, Zeiss, Oberkochen, Germany), atomic force microscopy (AFM; XE-70, Park Systems, Suwon, Republic of Korea), X-ray photoelectron spectroscopy (XPS, Sigma Probe, Thermo VG Scientific, East Grinstead, UK), and scanning transmission electron microscopy (STEM; Titan Themis G2 60-300, Thermo Fisher Scientific Inc.). Samples for STEM analysis were prepared using a focused ion beam scanning electron microscope (FIB, Helios NanoLab, FEI, Thermo Scientific, Waltham, MA, USA). To protect the area of interest during ion beam milling, only platinum (Pt) coating was used. STEM micrographs were recorded in high-angular annular dark field (HAADF) mode and analyzed using energy dispersive spectroscopy (EDS) and electron energy loss spectroscopy (EELS) detectors.

### 2.4. Toxicity of the Functionalized Ti-6Al-4V Surfaces

The biocompatibility studies of Ti-6Al-4V samples functionalized with Au-PEG-VH on a murine NIH/3T3 fibroblasts cell line (ATCC^®^ CRL-1658™, kindly supplied by Prof. B. Mizrahi, Faculty of Biotechnology and Food Engineering, Technion-Israel Institute of Technology) were conducted using the 3-[4,5-dimethylthiazol-2-yl]-2,5 diphenyl tetrazolium bromide (MTT, 200 μL, 5 mg/mL, Sigma-Aldrich, St. Louis, MO, USA) metabolic assay. NIH/3T3 cells were seeded at a density of 0.5 × 10^6^ cells per well in 6-well plates with 1.5 mL RPMI 1640 medium (Sigma-Aldrich, St. Louis, MO, USA) and incubated with samples of different coating types for 24 h and 72 h. Untreated cells were considered 100% viable and used as a control. MTT (5 mg/mL in RPMI 1640 medium) was added for 2 h at 37 °C. Dimethyl sulfoxide (2 mL, Carlo Erba Reagents, Val de Reuil, France) was subsequently added to dissolve the formazan crystals. Cell viability was defined as absorbance values at a wavelength of 530 nm measured by a microplate UV−vis spectrophotometer (Multiskan GO, Thermo Fisher Scientific Oy, Vantaa, Finland) of samples compared to negative controls. The results are expressed as the mean ± standard deviation (S.D.) of four samples (*n* = 4).

## 3. Results and Discussion

### 3.1. Fabrication of Au NPs on Ti-6Al-4V Surface by Solid-State DW

**Figure 2:**
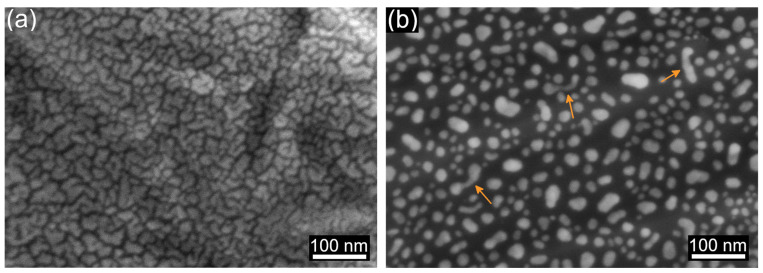
HR SEM micrographs of 5 nm-thick Au film on Ti-6Al-4V: (**a**) as-deposited, and (**b**) after annealing at 200 °C for 3 h. Arrows point to the NPs with a high aspect ratio.

The micrographs in Figure 2 show the surface of the Ti-6Al-4V alloy with the deposited Au film before (Figure 2a) and after solid-state DW (Figure 2b). An Au film of 5 nm in thickness was deposited to obtain fine NPs. The as-deposited Au film did not reach the “percolation threshold” (i.e., the minimum thickness required for the formation of a continuous film) and exhibited the morphology of densely packed clusters of complex shapes (Figure 2a). The Au clusters homogeneously covered the Ti-6Al-4V surface independently of the rough features underneath because the Au film thickness (5 nm) was significantly smaller than the typical size of the surface features (~5 µm).

After the annealing, we observed isolated Au NPs (Figure 2b)—a typical picture of fully dewetted thin film on flat surfaces [1]. Deposited Au clusters transformed into equiaxed NPs with a mean diameter of 19.8 ± 7.2 nm (Figure 2b). Similar to the as-deposited film, dewetted NPs homogeneously covered the Ti-6Al-4V surface. Occasional elongated NPs were present (Figure 2b, arrows); however, their lateral dimensions were close to the mean size of the equiaxed NPs. Moreover, closer observation of their shape unveiled that elongated NPs were composed of multiple grains, meaning that the selected DW conditions were insufficient for their full transformation into single-crystalline particles. Longer thermal treatments or higher temperatures will likely lead to a higher degree of equilibration of the elongated NPs. Interestingly, in the present work, the final stage of solid-state DW was achieved at a relatively low temperature of 200 °C. The reason for this is the discontinuous nature of the as-deposited film, limiting the diffusion distance on the surface of Au by the cluster size. Much thicker (20 nm) continuous Au films on amorphous and crystalline TiO_2_ did not reach the final solid-state DW stage, even after annealing at the temperature of 500 °C for 2 h [30].

We observed no correlation between the surface topography of the Ti-6Al-4V alloy and the shape or distribution of formed NPs (Figure 2b). Full DW was attributed to the discontinuity of the as-deposited film (Figure 2a) and nearly three orders of magnitude difference between film thickness and the typical size of the rough surface features. These observations are in good agreement with previously reported results on thin film DW on oxidized Ti-6Al-4V surfaces with natural roughness, where films thinner than the size of typical surface features undergo the usual steps of thin film DW on flat surfaces [25]. The present work confirms that surface roughness does not affect thin film DW when the film thickness is much smaller than the characteristic size of rough surface features.

**Figure 3:**
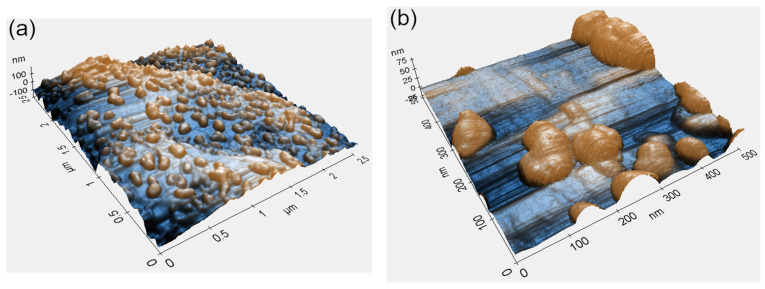
AFM topography micrographs of 5 nm-thick Au film on Ti-6Al-4V after annealing at 200 °C for 3 h at: (**a**) low, and (**b**) high magnification. Color code is linked to surface topography.

Figure 3 shows AFM topography micrographs of the Ti-6Al-4V surface with dewetted Au NPs acquired at different magnifications. Similar to the HR-SEM analysis results (Figure 2), these micrographs confirm full film agglomeration and its transformation into an array of separate NPs. No correlation between surface topography and shape or distribution of dewetted NPs was observed (Figure 3a). Minor differences in particle distribution (comparing Figure 3b to Figure 2b) most likely originate from local variations in deposited film thickness, a consequence of roughness irregularities. However, these variations were present at the submicron scale and did not influence the overall picture of the homogeneously distributed NPs (Figure 3a). The NPs exhibited some surface faceting showing that the particles evolve towards the equilibrium crystal shape (Figure 3b). However, the lack of prominent facets indicates that the time and temperature of DW treatment were insufficient to reach the full equilibration [31,32,33,34]. Given that thin film DW depends on film thickness, surface roughness, and heat treatment parameters [25,35,36,37,38], a thorough investigation of the dependence of size, shape, and distribution of the NPs on the above parameters should yield optimal processing conditions for a specific functional application.

### 3.2. Functionalization of the Ti-6Al-4V Surface with PEG-VH via Au NPs 

**Figure 4:**
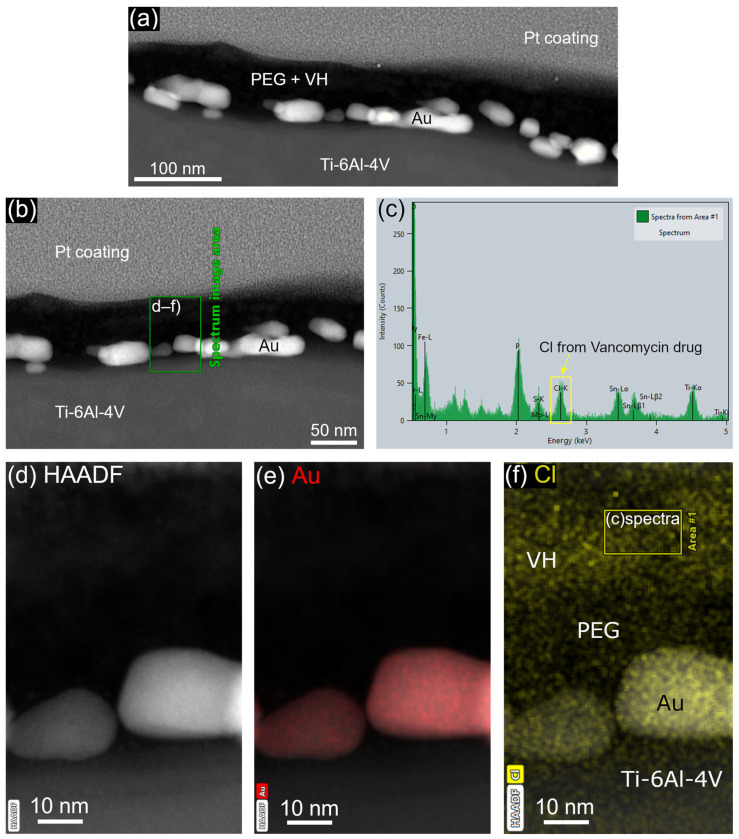
STEM cross-sectional micrograph of Ti-6Al-4V surface functionalized with Au-PEG-VH: (**a**,**b**) STEM HAADF micrograph and (**c**) EELS spectra of the selected area from (**f**), which highlights elemental Cl from VH drug; selected image area in (**d**) HAADF mode with elemental maps of (**e**) Au, (**f**) Cl. Pt coating—protective platinum coating.

**Figure 5:**
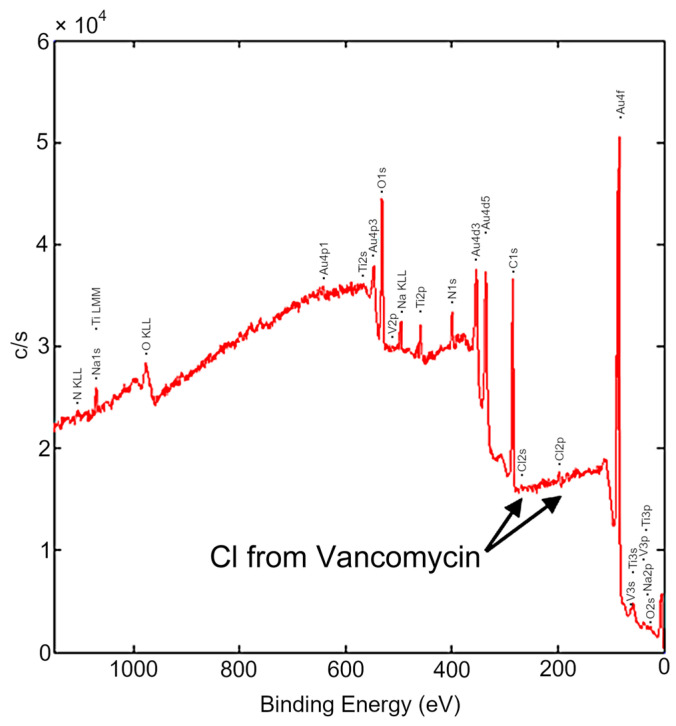
XPS spectrum of Ti-6Al-4V surface functionalized with Au-PEG-VH.

The conjugation of VH to the Ti-6Al-4V surface via DW-ed Au NPs with PEG linker was confirmed by STEM (Figure 4) and XPS (Figure 5) analyses. Drug conjugation was traced by chlorine (Cl) intrinsic to VH composition (Figure 4c,f and Figure 5). The Cl peak observed in the EELS data (Figure 4c) exhibited higher intensity in comparison to the peak observed in the XPS data (Figure 5). This disparity in peak intensity can be ascribed, in part, to the detection range of the two analytical methods. In the XPS technique, the detected electrons are derived from the uppermost few atomic layers near the surface, typically within a range of 5–10 nm [39]. Conversely, in the EELS technique, the spectrum is collected from a selected area, as depicted in Figure 4f. The selected thickness (depth) of the EELS area was set at about 10 nm, which can be up to twice the depth attainable by the XPS technique, resulting in the observed disparity in peak intensities. STEM images and an elemental map of the sample cross-section showed a continuous PEG layer covering Au NPs (Figure 4a,e,f). Continuous PEG film led to the formation of a continuous VH layer on the alloy surface (Figure 4a). The thickness of the VH layer measured from the elemental map was 18.8 ± 1.3 nm (Figure 4f).

**Figure 6:**
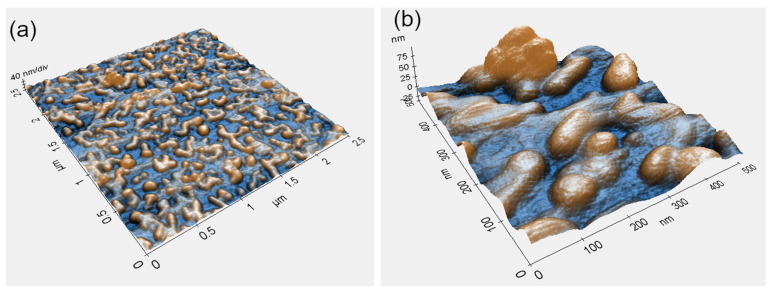
AFM topography micrographs (at different magnifications) of Ti-6Al-4V surface with DW-ed Au NPs after functionalization with VH at: (**a**) low, and (**b**) high magnification. Color code is linked to surface topography.

**Figure 7:**
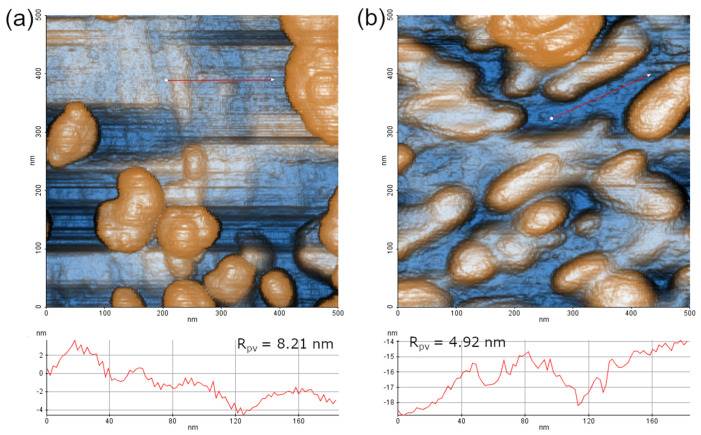
AFM topography micrographs of Ti-6Al-4V with DW-ed Au NPs: (**a**) before and (**b**) after functionalization with PEG-VH. The line topography profiles below the AFM images were acquired along the respective red arrows. Roughness R_pv_ is the sum of the maximum profile peak height (R_p_) and maximum profile valley depth (R_v_) of the corresponding line profile.

An additional, albeit indirect, confirmation of the VH layer formation was extracted from AFM scans of the surfaces before (Figure 3) and after (Figure 6) functionalization. Overall, the surface of Ti-6Al-4V and DW-ed Au NPs smoothened after functionalization (Figure 6): AFM scans of the initial sample show Au NPs and Ti-6Al-4V surface with sharp features (Figure 7a), which were visibly smoothened after the functionalization (Figure 7b). Ti-6Al-4V surface roughness R_pv_—a sum of maximum peak height (R_p_) and maximum valley depth (R_v_) of the scanned profiles—decreased from 8.21 nm to 4.92 nm after the VH film formation (line profiles, Figure 7). The decrease in surface roughness, coupled with the smoother topography line scans acquired between the particles, indirectly confirms the formation of a continuous functional layer between Au NPs. While STEM elemental mapping provides evidence of continuous film formation at the nanoscale (Figure 4), AFM scans and roughness measurements (Figure 7) illustrate the continuity of the film at the micrometer scale.

The developed approach to surface functionalization vial solid-state DW is simpler and more versatile compared to previously reported methods of Ti-based surface functionalization with VH by drop casting [40,41], cathodic electrophoretic deposition [42], and electrochemical deposition [43]. Moreover, it enables the fabrication of other metallic NPs, e.g., Ag, magnesium (Mg), iron (Fe), and zinc (Zn) employed in biomedical applications. Furthermore, the metal NPs produced by solid-state DW are exceptionally strong [44,45,46], which is required for mechanical stability during implant–bone interaction. Further investigations pertaining to mechanical stability, as well as chemical stability within physiological solutions, must be undertaken to elucidate the potential of the developed functional layer in the context of implantable applications.

### 3.3. Toxicity of Ti-6Al-4V Functionalized with Au-PEG-VH

**Figure 8:**
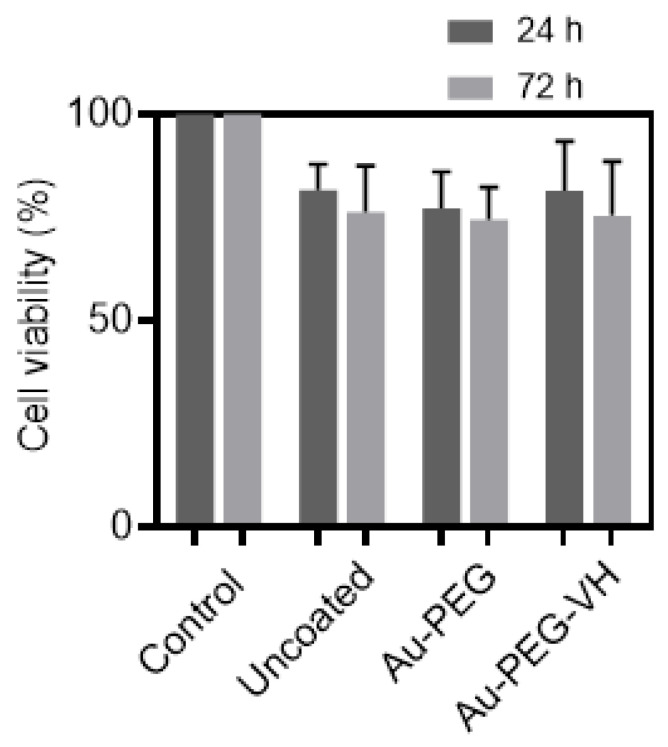
In vitro cell compatibility of Ti-6Al-4V samples functionalized with Au-PEG-VH using the NIH/3T3 fibroblasts cell line for 24 h and 72 h. Data are presented as the mean ± S.D. (*n* = 4).

The murine NIH/3T3 fibroblast cell line was used to evaluate the possible acute toxicity of Ti-6Al-4V samples functionalized with Au-PEG-VH. As shown in Figure 8, cell viability was above 70% for all samples tested. The decrease in cell viability is probably caused by cellular asphyxiation in the area below the samples, which can be clearly seen by comparing control of uncoated samples to their counterparts functionalized with Au-PEG-VH (Figure 8). Antibacterial activity of VH covalently bonded to the Ti-6Al-4V surface [47] and Ti beads [48] proved to reduce *Staphylococcus aureus* colony-forming in vitro. Based on that, we expect that the reported Ti-6Al-4V functionalized with Au-PEG-VH film will exhibit antibacterial activity.

## 4. Conclusions

In this work, we demonstrated a novel method for implant surface functionalization using Au NPs prepared by solid-state DW. We confirm the conjugation of antibiotic film of 18.8 ± 1.3 nm to Ti-6Al-4V surface via Au NPs by STEM and XPS analyses. Ti-6Al-4V surfaces coated with VH displayed relatively low musculoskeletal toxicity combined with a broad spectrum of activity against Gram-positive bacteria. The proposed approach of producing Au NPs for surface functionalization is simple, versatile, and can be adapted to the fabrication of other metallic NPs, e.g., Ag, Mg, Fe, and Zn, which can pave the way for the design of implant surfaces with novel functional properties.

## Data Availability

The original data associated with this manuscript can be obtained from the corresponding author (ER) upon reasonable request.
